# Species-Specific 5 mC and 5 hmC Genomic Landscapes Indicate Epigenetic Contribution to Human Brain Evolution

**DOI:** 10.3389/fnmol.2018.00039

**Published:** 2018-02-14

**Authors:** Andy Madrid, Pankaj Chopra, Reid S. Alisch

**Affiliations:** ^1^Department of Psychiatry, University of Wisconsin–Madison, Madison, WI, United States; ^2^Neuroscience Training Program, University of Wisconsin–Madison, Madison, WI, United States; ^3^Department Human Genetics, Emory University School of Medicine, Atlanta, GA, United States

**Keywords:** 5 mC, 5 hmC, brain evolution, monkey model, epigenetics

## Abstract

Human evolution from non-human primates has seen substantial change in the central nervous system, with the molecular mechanisms underlying human brain evolution remaining largely unknown. Methylation of cytosine at the fifth carbon (5-methylcytosine; 5 mC) is an essential epigenetic mark linked to neurodevelopment, as well as neurological disease. The emergence of another modified form of cytosine (5-hydroxymethylcytosine; 5 hmC) that is enriched in the brain further substantiates a role for these epigenetic marks in neurodevelopment, yet little is known about the evolutionary importance of these marks in brain development. Here, human and monkey brain tissue were profiled, identifying 5,516 and 4,070 loci that were differentially methylated and hydroxymethylated, respectively, between the species. Annotation of these loci to the human genome revealed genes critical for the development of the nervous system and that are associated with intelligence and higher cognitive functioning, such as *RELN* and *GNAS*. Moreover, ontological analyses of these differentially methylated and hydroxymethylated genes revealed a significant enrichment of neuronal/immunological–related processes, including neurogenesis and axon development. Finally, the sequences flanking the differentially methylated/hydroxymethylated loci contained a significant enrichment of binding sites for neurodevelopmentally important transcription factors (e.g., *OTX1* and *PITX1*), suggesting that DNA methylation may regulate gene expression by mediating transcription factor binding on these transcripts. Together, these data support dynamic species-specific epigenetic contributions in the evolution and development of the human brain from non-human primates.

## Introduction

The evolution of humans from non-human primates has seen substantial change in the central nervous system (Passingham, 2009), resulting in distinct differences in cognitive and behavioral capabilities. Humans and rhesus macaques share ~93% of their DNA sequences in the coding regions of the genome (Gibbs et al., 2007), suggesting that molecular mechanisms other than genetic mutations may contribute to their evolutionary diversity. Epigenetic modifications do not alter the underlying DNA sequence, yet are essential for the establishment and maintenance of transcriptional integrity throughout eukaryotic genomes. Thus, differences in human and non-human primate epigenetic landscapes may reveal key molecular substrates to their evolutionary divergence.

The most extensively studied epigenetic modification is the covalent addition of a methyl group to DNA, 5-methylcytosine (5 mC). In eukaryotic genomes, 5 mC often occurs at CpG dinucleotides and is associated with gene silencing. While eukaryotic genomes have a significant depletion of CpG sites throughout the genome, CpG-dense regions (CpG Islands) exist and are often found near the promoter regions of genes (Deaton and Bird, 2011). Previous studies comparing the 5 mC methylomes of humans and chimpanzees reported species-specific 5 mC levels were located in hundreds of genes that are associated with neurological disorders and cancer (Martin et al., 2011). The 5 mC levels on these genes were lower in gene promoter regions of humans compared to chimpanzees and were correlated to changes in gene expression (Zeng et al., 2012). Together, these data provided evidence for differential epigenetic landscapes in brain tissues from humans and non-human primates.

It recently was shown that 5 mC could be oxidized to a stable derivative, 5-hydroxymethylcytosine (5 hmC). While 5 mC is established and maintained by the family of DNA methyltransferases (DNMTs) (Smith and Meissner, 2013), 5 hmC is catalyzed by the ten-eleven translocation (TET) family of enzymes following exposure to environmental stimuli (e.g., oxidative stress; Ito et al., 2010). Interestingly, 5 hmC appears to have a brain-specific function, as it is enriched more than 10-fold in the brain compared to peripheral tissues, associates with the regulation of neuronal activity, and accumulates in the brain during neuronal development and maturation (Szulwach et al., 2011; Yao and Jin, 2014). Previous findings have identified that both 5 mC and 5 hmC levels are depleted on CpG Islands, with abundant levels found outside of CpG Islands (Ezura et al., 2009; Deaton and Bird, 2011; Chopra et al., 2014; Long et al., 2016; Lunnon et al., 2016). While a growing body of research has found that disruptions in 5 mC levels are associated with abnormal neurodevelopment and human disease (Irier and Jin, 2012; Pfeifer et al., 2013), others have reported that 5 hmC is independently associated to neurological disorders (e.g., Rett syndrome and Autism; Mellén et al., 2012; Zhubi et al., 2014; Papale et al., 2015) and neurodegenerative diseases (e.g., Huntington's and Alzheimer's; Chouliaras et al., 2013; Wang F. et al., 2013; Condliffe et al., 2014). Taken together, the above findings indicate that both 5 mC and 5 hmC are highly dynamic in the brain and that perturbations in these modifications contribute to brain development and function, as well as brain-related disorders.

The molecular mechanisms contributing to the evolution of complex neurodevelopmental processes from monkeys to humans remain unclear. Here, genome-wide profiles of 5 mC and 5 hmC levels in the brains of humans and non-human primates were examined, revealing species-specific DNA methylation and hydroxymethylation levels. Together, these data support unique roles for 5 mC and 5 hmC in neuronal-related processes associated with the evolution of the human brain.

## Results

### Humans and monkeys exhibit similar 5 mC and 5 hmC genomic landscapes

**Figure 1:**
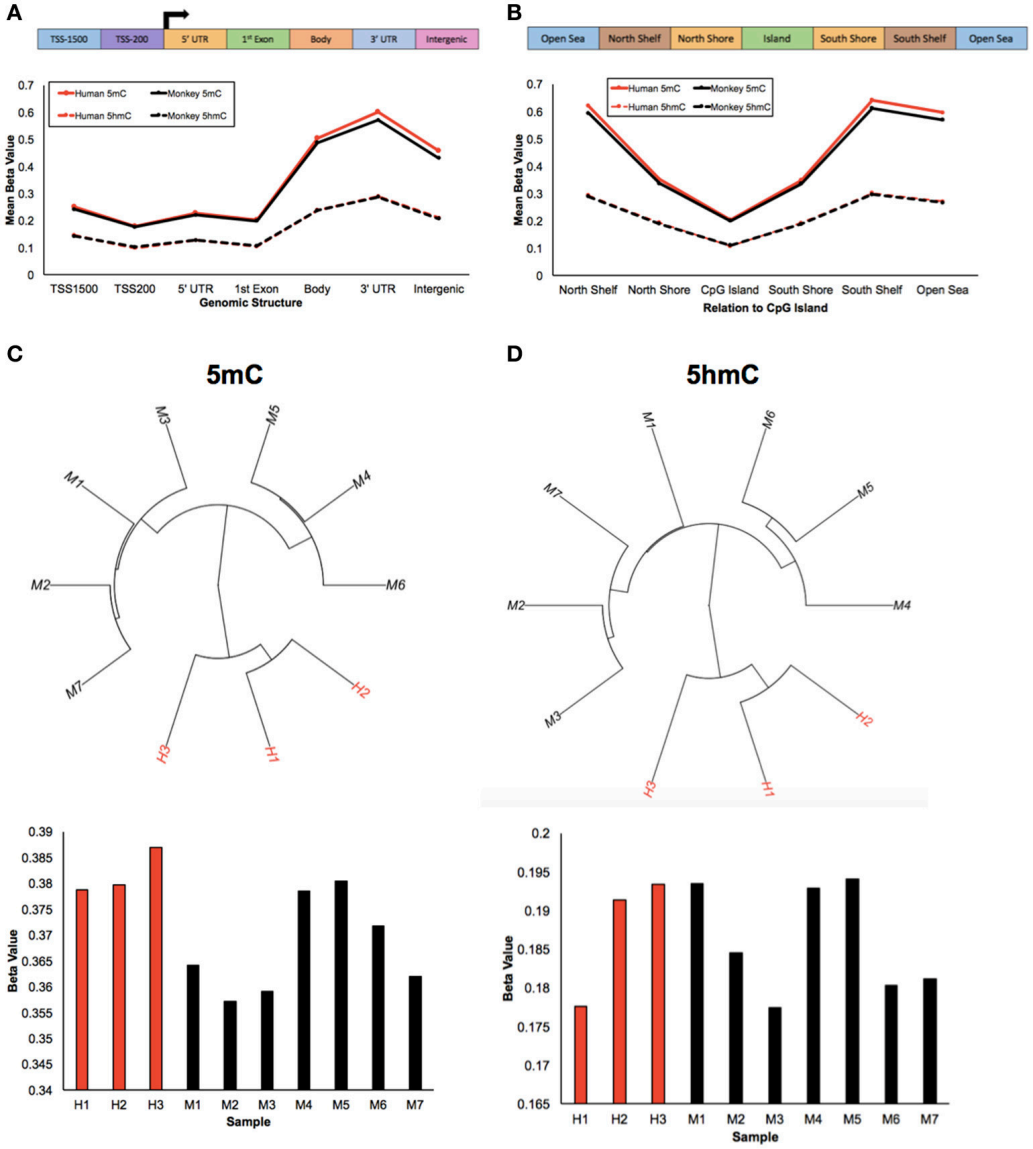
Humans and monkeys exhibit similar 5 mC and 5 hmC abundance trends. **(A)** Schematic of the standard genomic structures: 1,500 bp upstream of the transcription start site (TSS1500); 200 bp upstream of the transcription start site (TSS200); 5′ UTR; 1st exon; gene body; 3′ UTR; intergenic regions (top panel). Line plot of the mean beta value (y-axis) of 5 mC abundance from humans and monkeys (solid red and black lines, respectively) and 5 hmC abundance from humans and monkeys (dashed red and black lines, respectively) across standard genomic structures (x-axis; bottom panel). **(B)** Schematic of the structures in relation to CpG islands: North Shelf, North Shore, CpG Island, South Shore, South Shelf, Open Sea (top panel). Line plot of the mean beta value (y-axis) of 5 mC abundance from humans and monkeys (solid red and black lines, respectively) and 5 hmC abundance from humans and monkeys (dashed red and black lines, respectively) in relation to CpG Islands (x-axis; bottom panel). **(C,D)** Unsupervised hierarchical clustering results from 5 mC **(C)** and 5 hmC **(D)** beta values with human and monkey samples depicted in red and black, respectively (top panel), and the mean beta value (y-axis) from each 5 mC **(C)** and 5 hmC **(D)** sample (x-axis) (bottom panel).

To study epigenetic contributions in human brain evolution, 5-methylcytosine (5 mC) and 5-hydroxymethylcytosine (5 hmC) were profiled in brain tissue from humans and rhesus macaques (monkeys) using previously published HumanMethylation450 array data and a list of rhesus-competent probes (Chopra et al., 2014), which interrogated 142,304 5 mC loci and 140,664 5 hmC loci (Methods). As a first examination of the epigenetic landscapes between humans and monkeys, the mean methylation and hydroxymethylation levels of each species was plotted along standard genomic structures (Figure 1A; Methods). Consistent with previous comparisons with chimpanzees, both humans and monkeys had lower methylation levels in gene promoter regions and higher methylation levels within gene bodies and 3′ untranslated regions (UTRs) (Meyer et al., 2012; Zeng et al., 2012; Yang et al., 2014). Moreover, while hydroxymethylation levels overall were lower than methylation levels, the abundance of 5 hmC showed similar trends between humans and monkeys: lower 5 hmC levels in promoter regions and higher 5 hmC levels within gene bodies and 3′ UTRs. Together, these data suggest that like 5 mC the abundance of 5 hmC is evolutionarily conserved between humans and monkeys along standard genomic structures.

The mean abundances of 5 mC and 5 hmC for each species were next calculated in relation to CpG Islands (Figure 1B; Methods). Again, human and monkey methylation profiles were evolutionarily conserved; lower methylation levels in CpG Islands and higher methylation levels outside of CpG Islands, which supports previous comparisons with chimpanzees (Ezura et al., 2009; Deaton and Bird, 2011; Chopra et al., 2014; Long et al., 2016). While mean hydroxymethylation levels continued to be lower than methylation levels, they again showed similar dynamics to 5 mC: lower 5 hmC levels in CpG Islands and higher levels outside of CpG Islands. Together, these data further support that the DNA methylation genomic landscapes are evolutionarily conserved in humans and monkeys.

Despite these similarities of genomic structure profiles between species, we next sought to determine if there is information within the 5 mC and 5 hmC data that could distinguish these two species. Unsupervised hierarchical clustering analyses revealed distinct clusters of humans and monkeys using either genome-wide 5 mC or 5 hmC levels (Figures 1C,D). Together, these findings suggest that while humans and monkeys have similar epigenetic profiles in relation to standard genomic structures, 5 mC and 5 hmC levels are species-specific.

### Methylome and hydroxymethylome differences between humans and monkeys

**Figure 2:**
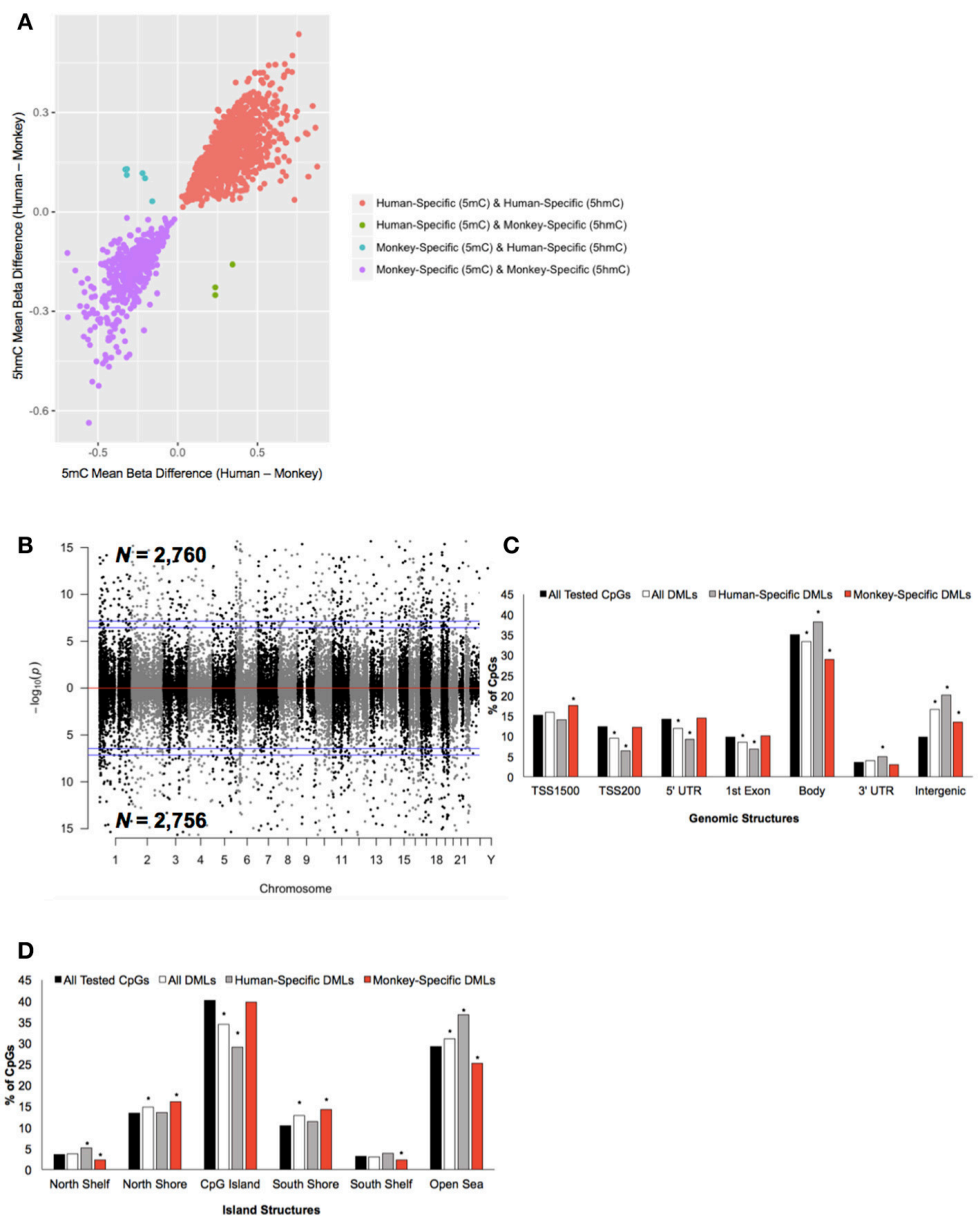
Characterization of DMLs across standard genomic structures. **(A)** Scatterplot of CpGs found to be both differentially methylated and differentially hydroxymethylated. The difference in the mean beta values from 5 mC (x-axis) and 5 hmC (y-axis) are plotted (positive value = more 5 mC/5 hmC abundance in humans and a negative value = more 5 mC/5 hmC levels in monkeys). Loci that were human-specific (red) and monkey-specific (purple) in both the 5 mC and 5 hmC datasets are shown. Loci that were human-specific in the 5 mC and monkey-specific in the 5 hmC (green) or monkey-specific in the 5 mC and human-specific in the 5 hmC (blue) are shown. **(B)** Modified Manhattan plot of species-specific DMLs from brain tissue reveals DMLs to be distributed across the entire human genome. Human-specific and monkey-specific DMLs are displayed with the –log10 of the raw *P*-value (y-axis). Significant DMLs are displayed outside of the dashed lines (genome-wide significance of 0.05 and 0.01), and they alternate between black and gray to indicate each chromosome. **(C)** The percent distribution (y-axis) of all CpGs investigated (black), all DMLs (white), human-specific DMLs (gray), and monkey-specific DMLs (red) in each genomic structure are shown. Significant over- and under-representation of DMLs are indicated (*P < 0.01). **(D)** The percent distribution (y-axis) of all CpGs investigated (black), all DMLs (white), human-specific DMLs (gray), and monkey-specific DMLs (red) in each island structure are shown. Significant over- and under-representation of DMLs are indicated (*P < 0.01).

To determine the extent of species-specific 5 mC and 5 hmC levels, differential methylation and hydroxymethylation analyses were conducted on these human and monkey DNA methylation data (Chopra et al., 2014). Notably, sodium bisulfite treatment alone cannot distinguish between 5 mC and 5 hmC; thus, resulting sodium bisulfite detected methylation levels represent a composite of 5 mC + 5 hmC levels. However, TET-assisted sodium bisulfite conversion of DNA allows for the sole detection of 5 hmC, making it possible to distinguish between differential 5 mC and 5 hmC levels by comparing the data from both sodium bisulfite and TET-assisted sodium bisulfite treated DNA. For loci found to be both differentially methylated and differentially hydroxymethylated, we examined whether the differential signal was contributed by 5 mC or 5 hmC (Methods). This analysis revealed that the vast majority (>99%) of these dual differential loci had similar abundances of composite (5 mC + 5 hmC) and 5 hmC, indicating that the differential signal is contributed by significant discrepancies in 5 hmC levels, not 5 mC (Figure 2A). Thus, CpG dinucleotides that were found to be both differentially methylated and differentially hydroxymethylated were solely classified as differentially hydroxymethylated. This approach identified 5,516 differentially methylated loci (DMLs) between humans and monkeys, which were distributed across the entire genome (aLIS *P* < 0.01; Figure 2B; Data Sheet 1; Methods). Annotation of the DMLs to standard genomic structures revealed, by permutation testing, significant enrichments and depletions across these structures. While DML over-representation was significant in intergenic regions, under-representation was significant in the TSS200, 5′ UTR, 1st exon, and within the gene body (Permutation *P* < 0.01; Figure 2C), suggesting more 5 mC differences outside of genes than would be expected by chance alone. DMLs were next filtered based on species-specific 5 mC levels: greater 5 mC in humans (i.e., human-specific DML; *N* = 2,760) or in monkeys (i.e., monkey-specific DML; *N* = 2,756). Human-specific DMLs were significantly over-represented in the gene body, 3′ UTR, and intergenic regions, while significantly under-represented in the TSS200, 5′ UTR and first exon (Permutation *P* < 0.01; Figure 2C). On the other hand, monkey-specific DMLs were significantly over-represented in the TSS1500, while being significantly under-represented in the gene body and intergenic regions (Permutation *P* < 0.01; Figure 2C). Together, these data indicate that human-specific differential 5 mC levels are depleted near the 5′ end of genes and enriched near the 3′ end of genes.

The DMLs were next annotated to CpG Islands and were found to be significantly over-represented in the north and south shores of CpG Islands and further than 4 kb outside of CpG Islands (open sea) and significantly under-represented on CpG Islands (Permutation *P* < 0.01; Figure 2D). Human-specific DMLs were significantly over-represented in the north shelves and the open sea, and significantly under-represented on CpG Islands (Permutation *P* < 0.01; Figure 2D). On the other hand, monkey-specific DMLs were significantly over-represented in the north and south shore and significantly under-represented in north and south shelves, and the open sea (Permutation *P* < 0.01; Figure 2D). Together, these data highlight species-specific profiles in relation to CpG islands, particularly the significant depletion of human-specific changes on CpG Islands. Since methylation levels on CpG Islands are linked to gene expression, these findings may reflect sites of species-specific gene expression.

**Figure 3:**
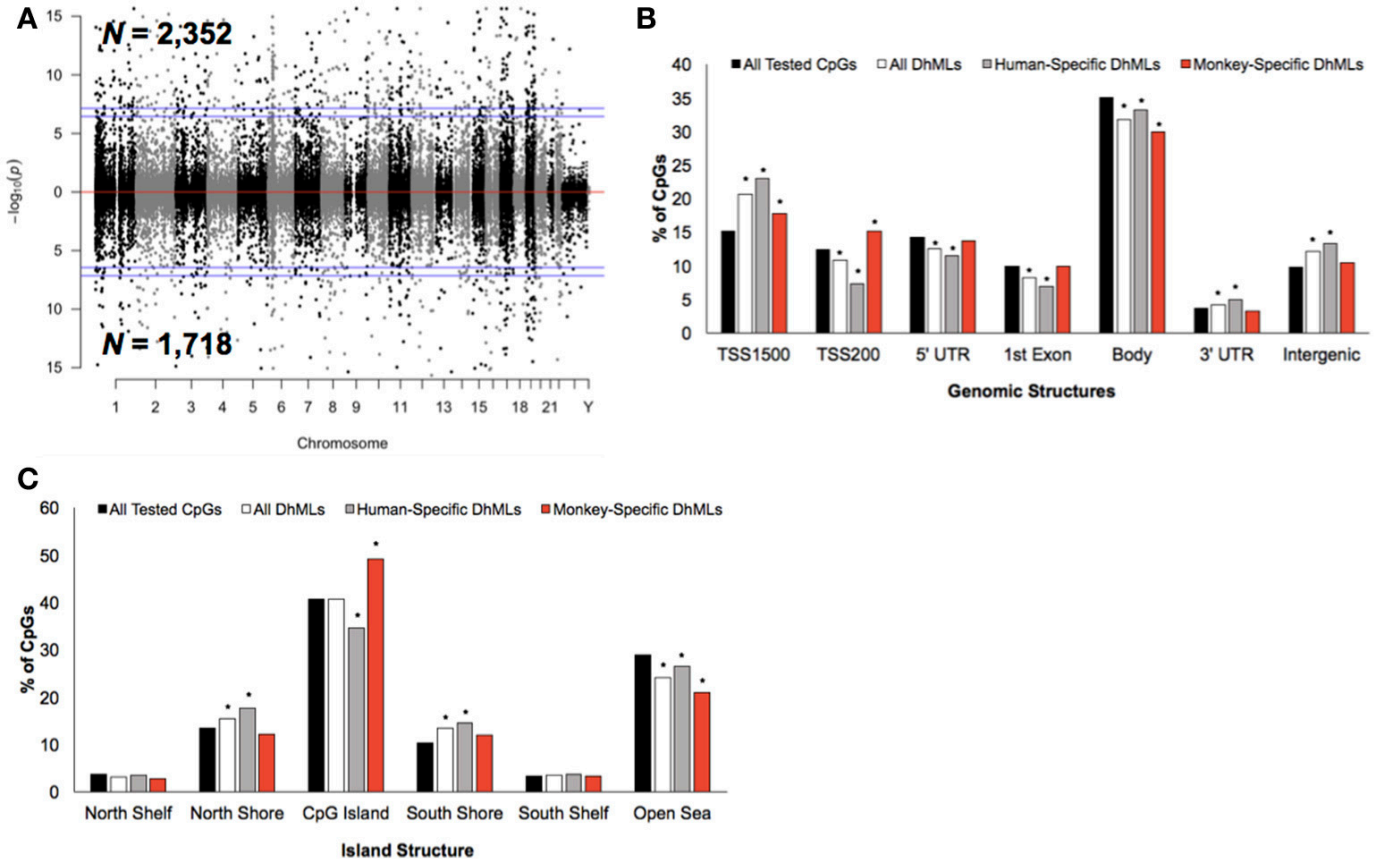
Characterization of DhMLs across standard genomic structures. **(A)** Modified Manhattan plot of species-specific DhMLs from brain tissue reveals DhMLs to be distributed across the entire human genome. Human-specific and monkey-specific DhMLs are displayed with the –log10 of the raw *P*-value (y-axis). Significant DhMLs are displayed outside of the dashed lines (genome-wide significance of 0.05 and 0.01), while DhMLs alternate between black and gray to indicate each chromosome. **(B)** The percent distribution (y-axis) of all CpGs investigated (black), all DhMLs (white), human-specific DhMLs (gray), and monkey-specific DhMLs (red) in each genomic structure are shown. Significant over- and under-representation of DhMLs are indicated (*P < 0.01). **(C)** The percent distribution (y-axis) of all CpGs investigated (black), all DhMLs (white), human-specific DhMLs (gray), and monkey-specific DhMLs (red) in each island structure are shown. Significant over- and under-representation of DhMLs are indicated (^*^P < 0.01).

Differential analysis of 5 hmC profiles revealed 4,070 differentially hydroxymethylated loci (DhMLs) between humans and monkeys, which were distributed across the entire genome and included 2,352 and 1,718 human-specific and monkey-specific DhMLs, respectively (aLIS *P* < 0.01; Figure 3A; Data Sheet 2; Methods). Permutation testing revealed significant DhML over-representation in the TSS1500, 3′ UTR, and intergenic regions and under-representation in the TSS200, 5′ UTR, 1st exon, and gene body (Permutation *P* < 0.01; Figure 3B). More specifically, human-specific DhMLs were significantly over-represented in the TSS1500, 3′ UTR, and intergenic regions and significantly under-represented in the TSS200, 5′ UTR, 1st exon, and gene body. Monkey-specific DhMLs were significantly over-represented in the TSS1500 and TSS200 and significantly under-represented in the gene body (Permutation *P* < 0.01; Figure 3B). Similar to the 5 mC data, these 5 hmC data indicate that human-specific DhMLs are depleted near the 5′ end of genes and monkey-specific DhMLs are depleted near the 3′ end of genes, which provides further evidence that may suggest important differences in species-specific regulation of gene expression.

In relation to CpG Islands the DhMLs were significantly over-represented in the north and south shore and under-represented in the open sea (Permutation *P* < 0.01; Figure 3C). Similarly, human-specific DhMLs also significantly over- and under-represented on these same structures, except human-specific DhMLs were significantly under-represented on CpG Islands. Monkey-specific DhMLs were significantly over-represented on CpG Islands and significantly under-represented in the open sea (Permutation *P* < 0.01; Figure 3C). Again finding a significant depletion of only human-specific changes on CpG Islands suggests that 5 mC and 5 hmC levels on human CpG Islands are lower compared to monkeys, which may directly relate to species-specific gene expression that could be important for species-specific brain functions.

### Differentially methylated and hydroxymethylated genes have neuronal functions

**Figure 4:**
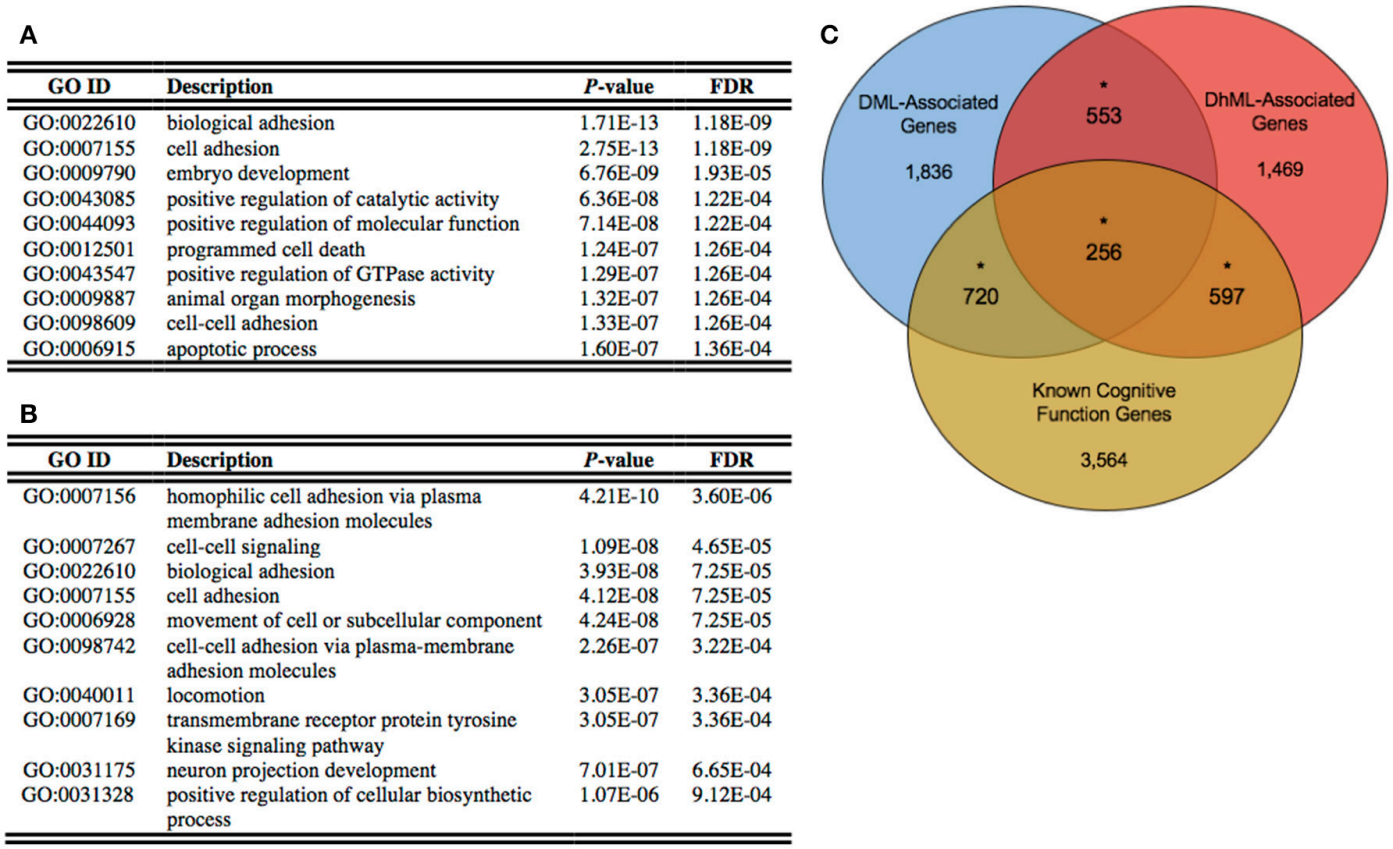
Differentially methylated and hydroxymethylated genes have neuronal functions. **(A)** Top ten gene ontological terms associated with all differentially methylated genes **(B)** Top ten gene ontological terms associated with all differentially hydroxymethylated genes **(C)** Venn diagram of the overlap of DML-associated genes (blue; *N* = 3,365), DhML-associated genes (red; *N* = 2,875) with genes known to function in intelligence, cognition, and learning and memory similarly tested within the gene universes (yellow; *N* = 5,137). The significant overlaps are indicated by an asterisk (Chi-square *P* < 0.05).

Annotation of the differentially methylated and hydroxymethylated loci to genes yielded 3,365 and 2,875 genes that were differentially methylated and hydroxymethylated, respectively, with 809 genes having both differential methylation and hydroxymethylation at different locations within the same gene (Data Sheets 1, 2). To gain insight into the relationships between the DML- and DhML-associated genes, separate ontological analyses were conducted on each gene set, revealing significant enrichments of neuronal/immunological-related terms in both data sets, including neurogenesis, axonogenesis, and neuron development (Chi-square *P* < 0.001; Figures 4A,B; Data Sheets 3, 4; Geifman et al., 2010). Together, these data suggest that differential 5 mC and 5 hmC are marking important genes related to neurological processes that may have contributed to the evolution of the human brain.

Identifying the neuronal enrichment of ontological terms prompted a comparison of the DML- and DhML-associated genes with a list of genes known to function in cognitive function (Methods). Both comparisons yielded significant overlaps: known cognitive function genes with DMLs (*N* = 976 of 5,137; Chi-square *P* < 0.001); and known cognitive function genes with DhMLs (*N* = 853 of 5,137; Chi-square *P* < 0.001; Figure 4C). These data further support that 5 mC and 5 hmC independently contribute to the evolution of human intelligence/cognition.

### Enrichment of sequence motifs near differentially methylated and hydroxymethylated loci

**Figure 5:**
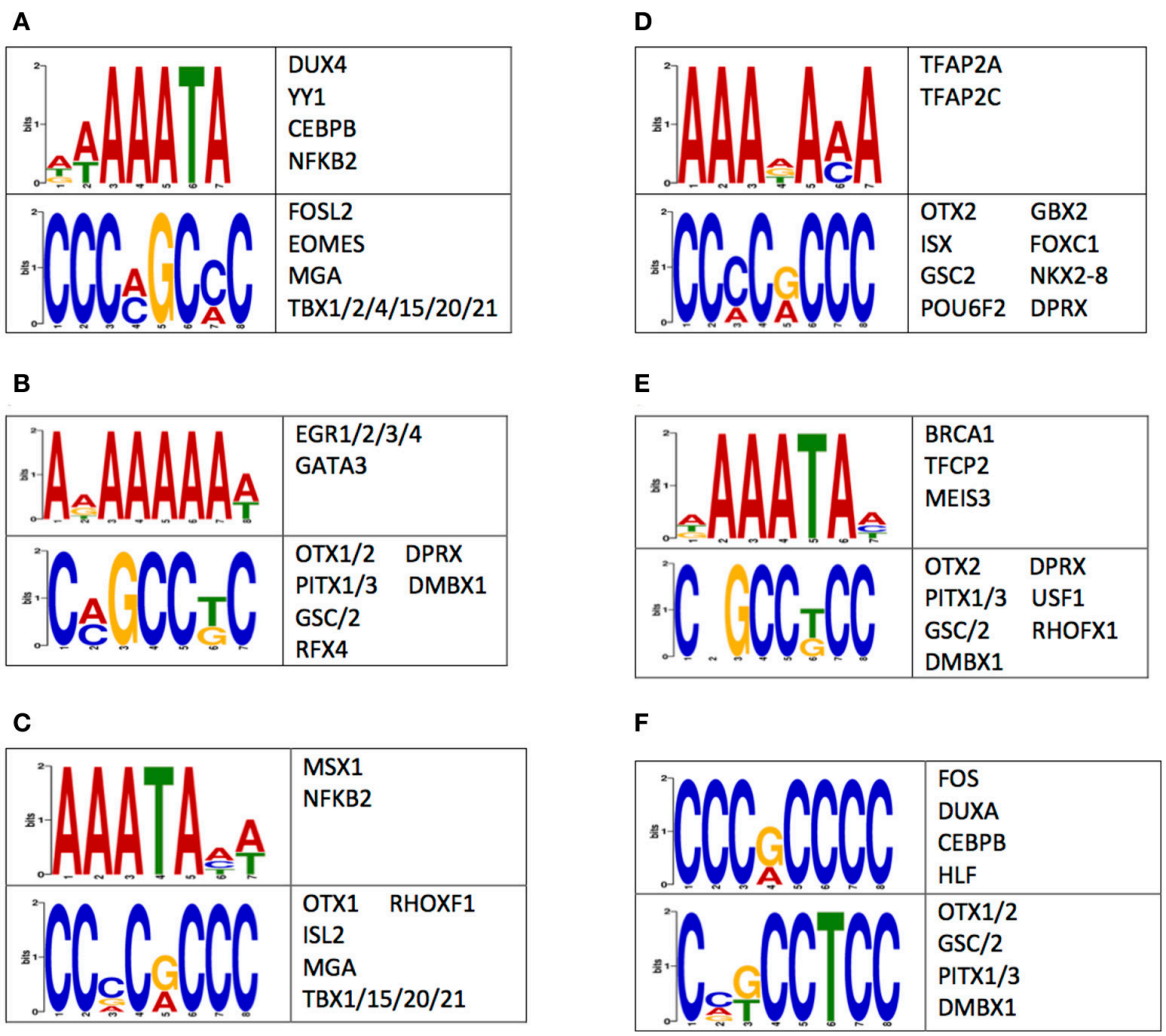
Sequences flanking DMLs and DhMLs contain putative transcription factor binding sites. The logo of enriched motifs found using sequences from **(A)** all DMLs, **(B)** human-specific DMLs, **(C)** monkey-specific DMLs, **(D)** all DhMLs, **(E)** human-specific DhMLs, and **(F)** monkey-specific DhMLs that were predicted by the DREME suite (*E* < 1e-4) are depicted. A table of transcription factors predicted to bind to these motifs are also shown.

A possible mechanism for the observed differences in 5 mC and 5 hmC may reside in the regulation of gene expression (Breiling and Lyko, 2015). Thus, the nucleotides flanking differentially methylated or hydroxymethylated loci were examined for enrichments of sequence motifs targeted by transcription factors (Methods). This examination revealed an enrichment of transcription factor binding sequence motifs (Figure 5). Notably, several of the transcription factors that bind to the enriched sequences have known roles in central nervous system development, such as *OTX1 OTX2, PITX1, NKX2-8*, and *NFKB2* (Frantz et al., 1994; Szeto et al., 1999; Bhakar et al., 2002; Philippi et al., 2007; Safra et al., 2013; Blank and Prinz, 2014). Together, these data support differential 5 mC and 5 hmC landscapes between humans and monkeys may have contributed to the evolution of the human brain by altering the binding affinity of neuronally-important transcription factors.

## Discussion

Here 5-methylcytosine (5 mC) and 5-hydroxymethylcytosine (5 hmC) profiles were investigated from human and non-human primate brain tissue to gain insight into the epigenetic differences between these two evolutionarily distinct species, related to the development of the human brain. Species-specific disruptions were found on genes known to be critical for the development of the nervous system, which was corroborated by significant enrichments of neuronal–related processes following ontological analyses. In addition, the DNA sequences flanking the differentially methylated/hydroxymethylated loci contained a significant enrichment of binding sites for neurodevelopmentally important transcription factors. Together, these data support dynamic species-specific epigenetic contributions in the evolution of the human brain.

This study revealed that both humans and monkeys grossly display similar 5 mC and 5 hmC profiles across the entire gene body and in relation to CpG Islands. Significant under-representations of DMLs and DhMLs were identified within promoter regions of genes and in regions proximal to CpG Islands in both humans and monkeys. These findings are similar to previous comparisons between humans and chimpanzees (Zeng et al., 2012), which also identified conservation of DNA methylation (the composite level of 5 mC + 5 hmC) profiles across standard genomic structures; lower methylation in the promoter region of genes and greater methylation within the gene bodies in all of these species (Zeng et al., 2012). In addition, humans exhibit significantly less methylation in the promoter region of genes compared to rhesus macaques, which is similar to what was observed when comparing human and chimpanzee composite methylomes (Zeng et al., 2012). Finally, the significant under-representation of DMLs and DhMLs along CpG Islands near gene promoters was predominantly due to significantly lower levels of *human*-specific DMLs/DhMLs, again supporting the hypothesis that differential methylation/hydroxymethylation of human promoters may result in differential gene expression of genes important for the evolution of the human brain. Together, these findings advocate that differential methylation and hydroxymethylation between humans and monkeys show significant discrepancies in their distributions across genomic structures that are associated with the regulation of gene expression, including CpG Islands, suggesting these distinct epigenetic landscapes result in species-specific modulation of gene expression.

Species-specific differences in 5 mC and 5 hmC abundance were found on several biologically relevant genes that are critical for brain development and functioning. For example, *RELN* was differentially hydroxymethylated among these species. *RELN* functions during development by facilitating cell-cell interactions and the migration of neurons throughout the developing central nervous system (Rice and Curran, 2001; Chao et al., 2009; Palmesino et al., 2010). In addition, *RELN* functions in synaptic plasticity, the maintenance of long-term potentiation, neurogenesis, and dendritic spine development in the adult brain (Weeber et al., 2002; Pujadas et al., 2010; Rogers et al., 2011). Similarly, *GNAS* was found to be differentially methylated between humans and monkeys. Imprinting of *GNAS* within the central nervous system is critical for metabolic regulation and processes governed by the central nervous system, such as postnatal growth and bone development (Eaton et al., 2012). Thus, these data suggest that species-specific differences in DNA methylation contribute to the regulation of biological processes such as development of the central nervous system.

Sequence motif discovery identified enrichments of sequences flanking the differentially methylated/hydroxymethylated loci. Several transcription factors that putatively bind within these sequences have known roles in the development of the central nervous system. For example, transcription factors *OTX1/2* were shown to help define regions and layers of cerebral cortex and of the cerebellum (Frantz et al., 1994). The cerebral cortex plays key roles in processes such as memory, learning, language, and awareness (Bear, 1996; Harasty et al., 1997; Grossberg, 2003; Merker, 2007). Thus, changes in the binding affinity of transcription factors that help to define cerebral cortical regions may allow for the higher thinking capacities and increased intelligence of humans. Additionally, the transcription factor *PITX1*, which has known mutations associated with pituitary development and the development of autism (Szeto et al., 1999; Philippi et al., 2007), putatively binds to sequences immediately flanking DMLs, suggesting that differential binding of *PITX1*, as a consequence of differential 5 mC and 5 hmC levels, may have contributed to the evolution of the pituitary gland. Other identified transcription factors, *NKX2-8* and *NFKB2*, are crucial for neural tube formation (Safra et al., 2013), myelination within the central nervous system (Blank and Prinz, 2014), and neuronal survival (Bhakar et al., 2002). Thus, evolutionarily distinct 5 mC and 5 hmC levels may alter the expression of genes related to these processes. It is of high interest to determine if differences in methylation and hydroxymethylation levels can alter transcription factor binding affinity and result in differences in the expression of neuronally-critical genes, especially those related to the evolution of the human brain.

The generation of species-specific datasets of both 5 mC and 5 hmC provides the unique opportunity to study the distinct profiles of these modifications in parallel, underscoring the importance of separately examining each of these DNA methylation modifications. The findings from this study suggest that previous studies using only sodium bisulfite methodologies to detect DNA methylation, which provides a single composite value of 5 mC + 5 hmC levels, and solely attributing their findings to 5 mC may need reinterpretation. Recently several studies have reported distinct molecular functions for 5 mC and 5 hmC in various tissues, particularly those within the central nervous system. This study is the first to implement differential 5 hmC analyses in an evolutionary context, which in turn provides an accurate report of differential 5 mC between human and non-human primate brain tissue.

While the approach employed here parallels that of other studies examining epigenetic differences between humans and non-human primates, aimed at gaining insight into the molecular evolution of human brain development (Martin et al., 2011; Zeng et al., 2012), this study uniquely profiles both 5 mC and 5 hmC. Future studies will functionally test whether species-specific genomic landscapes of 5 mC and/or 5 hmC can disrupt transcription factor binding of neuro-related transcription factors and determine the impact that this disruption has on gene expression. Indeed our knowledge of the molecular components influencing human brain evolution is still in its infancy; this study introduces new insights that implicate distinct roles for 5 mC and 5 hmC, providing evidence for epigenetic contributions in the evolution of the human brain.

## Methods

All data used here were previously generated and published (Chopra et al., 2014). Specific information regarding brain tissues examined and methods of sodium bisulfite and Tet-assisted sodium bisulfite (TAB) conversion of DNA are available in the published work. All experiments were approved by the University of Wisconsin—Madison Institutional Animal Care and Use Committee.

### Selection of CPGS for differential methylation and hydroxymethylation analysis

A previous publication identified that both 5 mC and 5 hmC abundances could be studied using the HumanMethylation450 array, using both human and non-human primate brain tissue samples (Chopra et al., 2014). Notably, other studies have found that 5 hmC abundance can be reliably and accurately measured utilizing the HumanMethylation450 array (Field et al., 2015; Sen et al., 2015; Johnson et al., 2016). As non-human primate DNA deviates from human sequences utilized in array probes, probes were first filtered using only those previously identified to contain at most four mismatches, reducing the number of probes investigated to ~154 k for both the 5 mC and 5 hmC datasets (Chopra et al., 2014). Beta values and detection *P*-values were next obtained from the same previous publication (Chopra et al., 2014). Notably, the previous publication from which data used for these analyses was gathered from used technical replicates for samples analyzed for 5 hmC abundance. As such, in order to incorporate as much data as possible, the average beta value for each tested CpG was generated for each pair of technical replicates and used for further downstream analysis. To further select CpGs with robustness for further analysis, beta values were converted to “NA” if the detection *P*-value exceeded 0.01 and CpGs were discarded if at most one sample was missing data (i.e., detection *P* > 0.01). This reduced the list of CpGs for analysis to 142,773 CpGs for the 5 mC dataset and 140,839 CpGs for the 5 hmC dataset.

### CpG annotation and hierarchical clustering

As an array-based method was utilized in this study, standard genomic structures and the relation to CpG Islands associated to each tested CpGs were obtained from the HumanMethylation450 annotation. The mean beta value for each species (i.e., Human of Monkey) was calculated for each CpG and was used to calculate the mean for each genomic structure using perl script. Unsupervised hierarchical clustering was performed in R environment using all beta values from all samples from all tested CpGs.

### Confounder adjustment

As multiple testing procedures utilized for DNA methylation data are often prone toward bias by latent confounding factors such as batch effects, R package *cate* (Wang et al., 2017) was employed to adjust for confounders by first estimating the number of confounders using a model where species was treated as the variable of interest and beadchip treated as a nuisance variable. Confounders were adjusted for using *cate* and achieved beta *P*-values were obtained for further analysis. Adjustment for confounders produced genomic inflation factors of 0.990 and 0.996 for the 5 mC and 5 hmC datasets, respectively. For the 5 mC dataset, this produced 21,377 CpGs with a raw *P* < 0.05, 9,999 CpGs that met a Benjamini-Hochberg cutoff <0.05, and 2,105 CpGs that met a Bonferroni correction < 0.05. For the 5 hmC dataset, these initial methods yielded 17,217 CpGs with a raw *P* < 0.05, 5,576 CpGs that met a Benjamini-Hochberg cutoff < 0.05, and 976 CpGs that met a Bonferroni cutoff < 0.05.

### Adjustment of local index of significance

As CpGs tested by array-based approaches share similar characteristics and correlations with nearby probes, a Hidden Markov Model was evoked to integrate prior knowledge in the detection of differential methylation and/or hydroxymethylation. To achieve this, CpGs were next ordered by chromosome and position, discarded if their *P*-value was returned as “NA” or equal to 0 or 1 from model fitting, and, for the remaining CpGs (5 mC: *N* = 142,304; 5 hmC: *N* = 140,664), *P*-values were transformed to *z*-scores. A Hidden Markov Model was used to adjust for local index of significance (aLIS) using R package *NHMMfdr* (Kuan and Chiang, 2012) with all parameters set to default with the exception of: model type set to HMM, alternative hypothesis type set to “kernel,” null hypothesis type set to empirical null with parameters set by estimated maximum likelihood, maximum iterations set to 100, epsilon value set to 1e-2. Finally, CpGs were considered significantly differentially methylated if their aLIS value was <0.01 (5 mC: *N* = 5,516; 5 hmC: *N* = 4,070). CpGs that were identified to be both differentially methylated and hydroxymethylated were investigated to examine if the differential signal was primarily contributed from 5 mC or 5 hmC abundance. First, for each locus, the mean beta value of 5 mC and 5 hmC for each species (i.e., human and monkey) was calculated. The mean beta value from monkey samples was subtracted from that of the human samples. The difference between the mean beta values of humans and monkeys, for both 5 mC and 5 hmC, were plotted against each other.

### Selection of model

Initially, a mixed-effects model using R packge *nlme* was employed and it should be noted that this method did show statistical bias, based on an identified genomic inflation factor of 2.01 for the 5 mC dataset and a genomic factor of 2.04 for the 5 hmC dataset. These large genomic inflation factors may result from the limited sample size used (5 mC: three human samples and seven monkey samples; 5 hmC: six human samples and 11 monkey samples) which is a limitation to this study. However, sample sizes used in this study are consistent with samples sizes from other publications investigating interspecies differential gene expression or DNA methylation (Khaitovich et al., 2005; Martin et al., 2011; Konopka et al., 2012; Zeng et al., 2012). In order to control for bias in these datasets, attempts were made to correct using the recently developed mechanisms, surrogate variable analysis using R package *sva* (Leek et al., 2012) and the adjustment of confounders using R package *cate* (Wang et al., 2017). No significant surrogate variables were identified in either the 5 mC or 5 hmC datasets using *sva. P*-values achieved through *sva* produced genomic inflation factors of 6.58 and 8.6 for the 5 mC and 5 hmC datasets, respectively. Thus, *sva* and *nlme* were unable to control the genomic bias. This led to the utilization of *cate* which was found to reduce the genomic bias to ~1 for each dataset. Therefore, beta *P*-values produced from *cate* after the adjustment for confounders were used for further analysis in the adjustment of local index of significance.

### Permutation testing of genomic structures and CpG Islands

Genomic structures and relation to CpG Islands associated to CpGs found to be differentially methylated or hydroxymethylated were obtained from the HumanMethylation450 array annotation. Notably, if a CpG was associated to more than one gene, along with being associated to more than one genomic structure, each genomic structure was separately used for further analysis. This generated 8,906 genomic structures for the 5 mC aLIS dataset and 6,967 genomic structures for the 5 hmC aLIS dataset. The number of times each genomic structure was observed in these datasets was calculated and termed the “actual-number.” The same numbers of genomic structures were randomly gathered from the full set of tested CpGs, and each genomic structure(s) associated to the tested CpGs, from the 5 mC and 5 hmC datasets (5 mC: *N* = 254,791; 5 hmC: *N* = 251,978), the number of times each genomic structure was tallied, termed the “permutated-number,” and compared to the “actual-number.” This method was repeated 10e5 times using perl script. The number of times the “permutated-number” exceeded the “actual-number” for each genomic structure was divided by 10e5, termed the permutated *P*-value for each genomic structure. For comparing the significant aLIS CpGs from the 5 mC dataset to those from the 5 hmC datasets to examine enrichments of genomic structures from each dataset, similar procedures were used above, yet the number of significant 5 mC CpGs was randomly selected from the significant 5 hmC CpGs datasets instead of the full datasets. *CpG Islands*: Much like for genomic structures, the “actual-number” of aLIS CpGs falling into each CpG Island structure was tallied and the same number of CpGs were randomly selected from the full datasets, the “permutated-number” found, tallied each time the “permutated-number” exceeded the “actual-number.” These procedures were conducted 10e4 times using perl script and divided by 10e5 to achieve permutated *P*-values.

### Gene and disease ontological analyses

Genes associated with DMLs or DhMLs were separately investigated for gene ontological enrichment of biological processes using WebGestalt (Wang J. et al., 2013) which utilized the human genome as the background reference set. An FDR cutoff of 0.05 was used to filter out gene ontology terms. Separate gene ontological analyses were utilized for genes associated to all DMLs, hyper-DMLs, hypo-DMLs, all DhMLs, hyper-DhMLs, and hypo-DhMLs. *Neuronal/immunological enrichment*: to identify if ontological findings showed a significant enrichment for neuronal/immunological-related terms, a Pearson's chi-square test with Yates' continuity correction was conducted in R using a published list of neuronal/immunological-related gene ontological terms (*N* = 3,071; Geifman et al., 2010). Notably, using the same methods described above we interrogated whether the HumanMethylation450 array gene universe had any bias for neuronal/immunological ontological terms and found no enrichment.

### Statistics for overlap with genes of known function(s)

Known intelligence-related genes: a chi-square test was used to compare DML- and/or DhML-associated genes, and genes tested in both gene universes that are known intelligence-related genes extracted from the GeneCards database using the following terms: intelligence; cognition; learning and memory (*N* = 5,137). Notably, the gene universes used for the chi-square test consisted of all the genes associated with tested CpGs after filtration in the 5 mC and 5 hmC datasets (5 mC: *N* = 19,077; 5 hmC: *N* = 19,038).

### Transcription factor motif discovery

The position of DMLs and DhMLs was taken and sequences ±250 bp of the DMLs was obtained for further processing using the human genome (hg19). Notably, if multiple DMLs were associated to the same gene and were less than 500 bp from each other, the sequences between these DMLs were clustered together for further analysis. The DREME suite (Bailey, 2011) was used to identify enrichments of transcription factor sequence motifs in the DML sequences using an *E*-value cutoff <1e−4. Putative binding factors were predicted using SpaMo directly from the DREME suite software package.

## Data access

The data generated from the monkey and human samples for this study can be found under the Gene Expression Omnibus (GEO) Gene Series: GSE49177.

## Author contributions

AM conceptualized the project, performed the analyses, and took part in writing of the manuscript. PC conceptualized the project and took part in the writing of the manuscript. RA conceptualized the project and took part in the writing of the manuscript.

### Conflict of interest statement

The authors declare that the research was conducted in the absence of any commercial or financial relationships that could be construed as a potential conflict of interest.
